# High performance work system and transformational leadership: Revisiting and questioning their implications for health-related wellbeing

**DOI:** 10.3389/fpsyg.2023.1072065

**Published:** 2023-02-09

**Authors:** Mats Ehrnrooth, Alexei Koveshnikov, Heidi Wechtler, Sven Hauff

**Affiliations:** ^1^Department of Management and Organization, Hanken School of Economics, Helsinki, Finland; ^2^Department of Management Studies, Aalto University, Espoo, Finland; ^3^Faculty of Business and Law, The University of Newcastle, New Castle, NSW, Australia; ^4^Department of Humanities and Social Sciences, The Helmut Schmidt University, Hamburg, Germany

**Keywords:** human resource management, leadership, job demands, job resources, wellbeing, emotional exhaustion, workload

## Abstract

Employee wellbeing represents a profound management challenge for both leaders and HR professionals, and both transformational leadership (TL) and high performance work system (HPWS) are assumed to play important roles in tackling this challenge. However, we know little about their unique and relative importance in promoting wellbeing. To shed light on this methodologically, theoretically and practically important issue, we draw mainly on leadership substitutes theory. Based on a comprehensive mediation model we examine whether HPWS substitutes the assumed relationships between TL and employee emotional exhaustion. Our study answers to three important calls for research: to examine the joint effects of leadership and HPWS, to examine their health-related impact, and to pursue more theory contesting research in management studies. Based on data from 308 white collar employees working under 76 middle-managers in five Finnish organizations our study points to the incompleteness of previous siloed research on both TL and HPWS, sheds new light on their relationships with wellbeing, and suggests ways to develop both TL and HPWS theory, thus providing important guidance for future research on their effects.

## 1. Introduction

Employees’ work-related wellbeing have profound individual, organizational and societal implications (Ford et al., 2011; Goh et al., 2016), and current research suggests that both leadership (Arnold, 2017; Harms et al., 2017; Inceoglu et al., 2018) and human resource management (HRM) (Van De Voorde et al., 2012; Peccei and Van De Voorde, 2019; Han et al., 2020) are fundamental drivers of wellbeing. However, one serious gap in our understanding of how to promote work-related wellbeing is due to the fact that research on leadership and HRM have advanced in separated research streams. Leroy et al. (2018) brought this fact into sharp focus and called for research simultaneously considering leadership and HRM to improve our understanding of “how to effectively manage people in organizations” (2018: 249). Similarly, other leadership researchers have argued that leadership and HRM represent distinct goal-directed influence processes, the important question being which of these inputs “do organizations need, and how do organizations develop and leverage the inputs for desired outcomes” (Fischer et al., 2017: 1,732). Research aiming to answer such questions with a focus on wellbeing is still very scarce (Kalshoven and Boon, 2012; Jo et al., 2019; Salas-Vallina et al., 2020; Wang et al., 2021; Hauff et al., 2022a,b).

In the present study, we focus on the unique and relative importance of a high performance work system (HPWS) and transformational leadership (TL) for emotional exhaustion. Emotional exhaustion refers to “feelings of being overextended and depleted of one’s emotional and physical resources” (Maslach and Leiter, 2008, p. 498). This is an important health-related outcome as it not only is a strong primary sign of burnout (Maslach and Leiter, 2008) but also has negative performance consequences (Demerouti et al., 2014). Following extant research, we consider TL as constituted by four leader behaviors: idealized influence, inspirational motivation, intellectual stimulation, and individualized consideration (e.g., Arnold, 2017). TL is the most studied form of leadership with extensive accumulated evidence of its relative importance for wellbeing-related employee attitudes in relation to other leadership styles (Hoch et al., 2018) but also with important remaining questions concerning its potential wellbeing “trade-off effects” (Inceoglu et al., 2018: 187). HPWS is the most studied form of HRM, with more controversial wellbeing outcomes (Han et al., 2020). Although still debated (e.g., Villajos et al., 2019), Boon et al. (2019) consider HPWS to be constituted by at least “six core HRM practices” (2019: 2,529): training, participation, compensation, performance evaluation, selection, and job design. Received knowledge in these highly influential research streams supports a negative relationship between both TL and emotional exhaustion (Harms et al., 2017) and HPWS and emotional exhaustion (Fan et al., 2014; Kloutsiniotis and Mihail, 2020; Wang et al., 2022), with some partially conflicting findings with respect to HPWS (Kroon et al., 2009; Zhang et al., 2013; Wang et al., 2022). Notably, this evidence is based on the respective siloed research streams and therefore runs a severe risk of being biased due to omitted variables (e.g., Hill et al., 2021). In order to address the bulk of previous research we focus on the full constructs of TL and HPWS, even as subdimensions of each have been shown to have differential effects (Arnold, 2017; Ogbonnaya and Messersmith, 2019).

In distinction to existing simultaneous research on leadership, HRM and wellbeing, which in line with Leroy et al. (2018) has mostly focused on their interaction effects (e.g., Salas-Vallina et al., 2020; Wang et al., 2021; Hauff et al., 2022a), our study is motivated by a relatively unexamined and provocative conjecture in the literature. based on leadership substitutes theory Jermier and Kerr (1997) suggested that “technological, structural, and other impersonal processes in the organization” are likely to substitute the main effects of pure interactional leadership behavior, thus challenging “the view that interpersonal leadership should be seen as the primary theoretical category” (1997: 98) in understanding organizational behavior. this conjecture deserves to be tested as also other scholars have argued that we may over-estimate the influence of leadership behavior (Meindl, 1995; Bligh et al., 2011). such tests are also important from a theory contesting perspective grounded in philosophy of science (Gray and Cooper, 2010; Leavitt et al., 2010) because both TL and HPWS, as we shall argue in more detail, purport to explain many of the same wellbeing related mechanisms and outcomes. HPWS clearly exemplifies an important organizational influence process whose main effects may substitute those of interpersonal leadership behavior, and which may also indirectly broaden our understanding of the mechanisms through which leaders, in distinction to leadership, can best influence organizational behavior (Jermier and Kerr, 1997). against this background we formulate our research question as follows: *Do the main effects of HPWS substitute the assumed main effects of TL on emotional exhaustion?*

In the present study the theoretical reasoning is thus based on the later interpretation of leadership substitutes theory (Jermier and Kerr, 1997) while the focal methodological approach is based on theory contests (Gray and Cooper, 2010; Leavitt et al., 2010). Specifically, to answer our research question we build on an important insight in the philosophy of the scientific method and follow the advice to test both the extent to which TL and HPWS “control the same variance” in emotional exhaustion, and whether HPWS adds “significant explained variance” (Leavitt et al., 2010: 655).

Based on a sample of 308 employees working under 76 middle-level managers in five Finnish organizations our study makes several important contributions. First, we extend previous simultaneous research on HRM and leadership which has mostly focused on their interaction effects (e.g., Salas-Vallina et al., 2020; Wang et al., 2021; Hauff et al., 2022a), by focusing on their main effects. Second, in so doing, we shed light on the independent importance of HPWS and TL, and specifically their under-researched direct and mediated associations with negative health-related wellbeing (Inceoglu et al., 2018; Han et al., 2020). The results point to the incompleteness of much of the hitherto siloed literatures on both TL and wellbeing (Arnold, 2017; Harms et al., 2017; Inceoglu et al., 2018) and HRM and wellbeing (Van De Voorde et al., 2012; Peccei and Van De Voorde, 2019; Han et al., 2020) and suggest the need for theoretical developments in both research streams. However, the results also corroborate some recent research suggesting positive effects of HRM on health-related outcomes (Fan et al., 2014; Kloutsiniotis and Mihail, 2020; Wang et al., 2022). Finally, our theory contest lends support for the proposition that conducting competitive tests between theories constitutes an important but neglected part of the “theory building process” (Gray and Cooper, 2010: 621). For practitioners, our research suggests that it is important to prioritize organizational investments of scarce resources in HPWS over TL if the goal is to improve health-related employee wellbeing.

## 2. The received knowledge of the impact of TL and HPWS

The job demands-resources (JD-R) model (Demerouti and Bakker, 2011) has been used to explain both the relationship between TL and wellbeing (Arnold, 2017) and that between HRM and wellbeing (Boxall et al., 2016; Van De Voorde et al., 2016). To argue for the *assumed* relationships in the respective siloed research streams we thus build on the integrative JD-R model, but only to establish the plausibility and importance of testing our hypotheses which focus on the substitution effects of HPWS.

In the JD-R model job demands is considered a primary source of stress, and a prime cause of emotional exhaustion, while various resources counteract this influence. Three central resources that according to the JD-R model can offset the negative effects of job demands and improve wellbeing are job control (e.g., Bakker et al., 2004), self-efficacy and organization-based self-esteem (Xanthopoulou et al., 2007). These resources reflect the three basic human needs, autonomy, competence and belongingness (Demerouti and Bakker, 2011). They are also good examples of the social-cognitive and motivational processes proposed to mediate the effects of leadership on negative health related outcomes such as exhaustion, more research on which is called for Inceoglu et al. (2018). Both TL and HPWS can be seen as overarching organizational resource providers, but with the potential to also increase job demands. In the case of TL, extant evidence largely suggests that its resource-based, exhaustion-reducing influence is reinforced by its stress-reducing effect (Arnold, 2017; Harms et al., 2017), with some indications of the opposite and related calls for more research probing this (Inceoglu et al., 2018). In contrast, an influential argument in the literature on HPWS is that its resource-based exhaustion-reducing influence will be counteracted by its work intensification effect that increases job demands and undermines wellbeing (Van De Voorde et al., 2012; Peccei and Van De Voorde, 2019).

In the following we thus first build on the JD-R model and research on TL and HPWS to identify their expected direct and mediated relationships with emotional exhaustion based on the received knowledge in the hitherto separated research streams. We then move to the focus of the paper, developing our competitive tests (Gray and Cooper, 2010) between TL and HPWS based on leadership substitutes theory (Jermier and Kerr, 1997). Our proposition is that the more structural and impersonal HPWS may offer an important alternative explanation (Leavitt et al., 2010) to that of interpersonal TL behaviors.

### 2.1. TL, job demands, job resources, and emotional exhaustion

A substantial body of research suggests that TL decreases work stress, strain, exhaustion and burnout (Arnold, 2017; Berger et al., 2019) and meta-analytic evidence supports the negative relationship between TL and both work stress and emotional exhaustion (Harms et al., 2017). The latter summarize the key arguments in the literature for these effects by saying that transformational leaders reduce ambiguity, provide encouragement and support, and allow followers to use their resources more effectively. Thus, previous research suggests that TL is negatively related to experienced job demands and positively related to resources and thereby also, in line with the JD-R model (Demerouti and Bakker, 2011), negatively related to emotional exhaustion (e.g., Fernet et al., 2015).

As for the effects of TL on our focal resources, TL theory and existing siloed empirical research also supports TL’s beneficial effects on all mediating resources, experienced job control/autonomy (Breevaart et al., 2014), self-efficacy (Gregersen et al., 2014; Arnold, 2017) and organization-based self-esteem (Kark et al., 2003).

**FIGURE 1:**
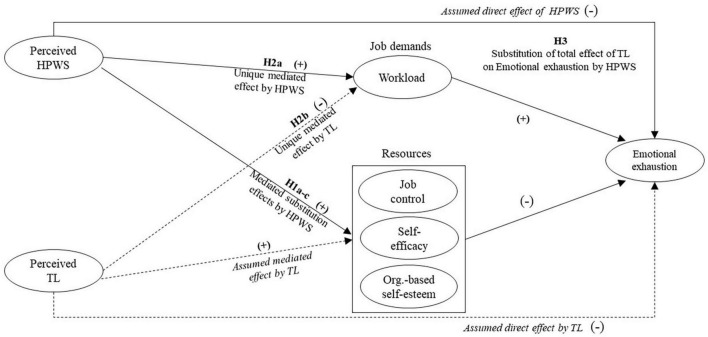
Assumed TL-only model with dotted lines. TL, transformational leadership; HPWS, high performance work system. The model depicts individual- level relationship.

Thus, despite some remaining questions about TL’s potential job demands increasing effect (Inceoglu et al., 2018) extant research largely suggests that TL reduces emotional exhaustion not only by reducing job demands, but also by increasing job control, self-efficacy and Organization-based self-esteem (OBSE). This leads us to put forth a model of the *assumed* relationships between TL and emotional exhaustion (denoted by dotted lines in Figure 1), notably based on existing research that has *not* accounted for the simultaneous influence of HPWS. This forms our baseline model in the analysis.

### 2.2. HPWS, job demands, resources, and emotional exhaustion

The question of job demands lies at the heart of what has come to be called the dark side of HRM, in particular of HPWS, i.e., its tendency to increase job demands with consequential stress-related negative effects on employee wellbeing (Jensen and Van De Voorde, 2016; Han et al., 2020). For example, Jensen et al. (2013) argue that research “suggests that HPWS…(is) resulting in role overload, burnout, and heightened pressure for individuals” (2013: 1,701). These effects have been theorized based on labor process theory (e.g., Ramsay et al., 2000) and a combination of AMO (ability, motivation, opportunity) theory, signaling theory, and identity theory (Ehrnrooth and Bjorkman, 2012). The key argument is that HRM/HPWS gives rise to increased work pressure due to its controlling effects. This controlling effect may take the form of what Ramsay et al. (2000) call “responsible autonomy” (2000: 505), or simply the fact that “the message that the system signals to the employees is one of increasingly higher performance” (Kroon et al., 2009: 512; Van De Voorde et al., 2016). In line with this, several studies have found evidence for a positive relationship between HPWS and job demands (Ramsay et al., 2000; Kroon et al., 2009; Jensen et al., 2013; Heffernan and Dundon, 2016; Ogbonnaya and Messersmith, 2019) and HPWS and stress (Qi et al., 2021; Zhu et al., 2022).

However, the evidence is still inconclusive for the negative effects of HPWS on work intensification, and in particular on emotional exhaustion, thus warranting further research on the topic. First, among the scarce research specifically on exhaustion, some found no direct main effect of HPWS (Kroon et al., 2009; Zhang et al., 2013; Wang et al., 2021), while Kloutsiniotis and Mihail (2020) recently found HPWS to be negatively related to exhaustion and, surprisingly, that this was mediated by the negative relationship between HPWS and job demands. Further, both Fan et al. (2014), Wang et al. (2022) found a similarly negative relationship between HPWS and burnout, closely related to exhaustion. Finally, these results may be explained by theory and evidence for the beneficial resource-based effects of HPWS, and specifically its effects on all the focal mediating resources in the present study, job control/autonomy (Wattoo et al., 2020), self-efficacy (Beltrán-Martín et al., 2017; Wattoo et al., 2020) and OBSE (Zhang et al., 2018; Wang et al., 2022). These resource-based motivational effects of HPWS should at least reduce exhaustion according to the JD-R model.

In summary, the evidence for the relationships of HPWS with both job demands and emotional exhaustion is inconclusive. On balance, however, both theory and evidence suggest that HPWS overall is negatively related to emotional exhaustion, particularly as mediated through the focal resources of job control, self-efficacy and OBSE, but positively related to job demands and thereby having a marginal counteracting effect that increases exhaustion.

Based on this received theoretical and empirical understanding of the effects of both TL and HPWS (see Figure 1), so far based on siloed research on both TL and HPWS, we now turn to the development of our hypotheses.

## 3. Hypotheses development

### 3.1. Theory contest based on leadership substitutes theory: HPWS as an alternative explanation of TL’s effects on emotional exhaustion

Theory contests between competing theories are crucially important in that they “can help establish which theory is superior in a given domain and force more precision in the theoretical arguments for the theory that fares less well” (Gray and Cooper, 2010: 629). While none of the theories used in either research on TL or HPWS helps us understand which theory is likely to be stronger, somewhat neglected leadership theory does offer interesting guidance. Recognizing the “phenomenological significance of leadership” Meindl argued that we may be prone to ““false assumption-making” regarding the relative importance of leadership factors to the functioning of groups and organizations” (1995: 330). While romance of leadership theory has focused specifically on understanding our biased attributions of causality to leaders (Meindl, 1995; Bligh et al., 2011), another way to question leadership as “the catch-all antecedent” to organizational outcomes (Bligh et al., 2011: 1,074), is to look for alternative explanations of outcomes that have been attributed to leadership. Leadership substitutes theory points us to such alternatives, also questioning the importance we attribute to leadership. In their later elaboration on the theory, Jermier and Kerr (1997) conjectured that organizational practices and processes may substitute the main effects of interpersonal leadership behavior, and thus offer an important alternative explanation of its assumed outcomes. Their proposition, which has remained largely untested, is that leaders exert much of their influence “through technological, structural, and other impersonal processes in the organization,” rather than through leadership behaviors understood as pure “superior-subordinate” interpersonal interactions (1997: 98). The effects of such behavioral interactions are what most existing leadership theories and styles are concerned with, including TL. In distinction to most extant research on leadership substitutes theory, their argument is that it is the main effects of organizational process substitutes of leadership that “deserve our attention” and that these may challenge the primary theoretical role of leader-centric interpersonal leadership behavior (1997: 98).

To put the above arguments and conjectures to a series of competitive tests (Gray and Cooper, 2010) we propose that HPWS offers an alternative explanation (Leavitt et al., 2010) to that of TL’s purely interpersonal and leader-centric explanation of emotional exhaustion. Such competitive tests are particularly relevant as TL and HPWS, as we have shown, are likely to exert their influence through the same focal JD-R-based mediating mechanisms. Based on this we hypothesize as follows (Figure 1).

Hypothesis 1a: The main effect of HPWS substitutes the negative main effect of TL on emotional exhaustion as mediated by job control/autonomy. Hypothesis 1b: The main effect of HPWS substitutes the negative main effect of TL on emotional exhaustion as mediated by self-efficacy. Hypothesis 1c: The main effect of HPWS substitutes the negative main effect of TL on emotional exhaustion as mediated by OBSE.

Further, as HPWS and TL are expected to have opposite effects on job demands, HPWS increasing them and TL decreasing them, we put forth the following hypotheses:

Hypothesis 2a: HPWS is positively related to emotional exhaustion as mediated by job demands. Hypothesis 2b: TL is negatively related to emotional exhaustion as mediated by job demands.

Finally, based on the arguments and evidence of a negative relationship between HPWS and emotional exhaustion we put forth our final and overall hypothesis as follows:

Hypothesis 3: The total negative main effect of HPWS substitutes the total negative main effect of TL on emotional exhaustion.

## 4. Materials and methods

### 4.1. Sample

We recruited five Finnish multinational companies from a pool of organizations that were either partner organizations to a major Finnish business school or had taken part in leadership development programs offered by the school. Our target was to gain access to 125 work units and 500 respondents. Five organizations signed up for the project and, with the help of a contact person in each organization who gathered voluntary participants whose anonymity was guaranteed, we received email addresses to 483 office workers. 308 employees from 76 work units in the five companies responded to our online survey (the employee response rate was 64%). In this dataset the respondents are thus nested within work units headed by a middle-level manager whose leadership behavior is evaluated by the employee-level respondents. On average the data consists of 4.1 subordinate respondents per work unit, with a mean age of 44 years and a mean tenure of 2.7 years working under the same middle-level manager in the same unit. 66% of the respondents were male and all respondents came from the capital region of Finland. All measurement instruments are exhibited in Appendix 1. Their psychometric properties are exhibited in Table 1 and their external validity is also evaluated below.

**TABLE 1:** Descriptive statistics, reliability scores, and correlations.

	Mean	Std. deviation	ICC	CR	AVE	DV	1	2	3	4	5	6	7	8	9
1. Emotional exhaustion	2.68	0.84	0.09	0.81	0.59	0.77	**0**.**66**	–	–	–	–	–	–	–	–
2. TL	3.41	0.69	0.18	0.9	0.5	0.71	-0.15	**0**.**88**	–	–	–	–	–	–	–
3. HPWS	23.24	3.91	0.18	−^a^)	−^a^)	−^a^)	-0.27	0.61	**0**.**85**	–	–	–	–	–	–
4. Job demands	3	0.84	0.07	0.86	0.68	0.82	0.61	0.1	-0.05	**0**.**76**	–	–	–	–	–
5. Job control	3.92	0.7	0.04	0.9	0.61	0.78	-0.14	0.24	0.38	-0.04	**0**.**87**	–	–	–	–
6. Self-efficacy	4.21	0.59	0.07	0.9	0.7	0.84	-0.04	0.19	0.27	0.06	0.39	**0**.**85**	–	–	–
7. OBSE	3.83	0.73	0.09	0.87	0.69	0.83	-0.13	0.42	0.54	0.11	0.4	0.62	**0**.**76**	–	–
8. Female	0.34	0.48	–	–	–	–	-0.02	-0.06	-0.03	0.04	-0.08	-0.08	-0.08	–	–
9. Age	43.71	10.1	–	–	–	–	0	-0.1	0	-0.08	-0.07	0.08	0.02	-0.1	–
10. Tenure with superior	2.71	2.99	–	–	–	–	0.07	-0.03	0.01	0.01	0.03	-0.09	-0.07	-1	0.16

ICC, intraclass correlations; CR, composite reliability; AVE, average variance extracted; DV, discriminant validity according to Fornell-Larcker criterion; a) Formative measurement construct. Cronbach’s alphas on the diagonal (bolded). All correlations above | 0.09| are significant at the 5% level.

### 4.2. Dependent variable

Emotional exhaustion was measured by the three best-loading items in Maslach and Jackson’s (1981) original measurement instrument for emotional exhaustion except for the item “I feel burned out from my work.” We excluded the latter because we did not expect all employees to be familiar with the term “burned out.” Cronbach’s alpha of the scale was 0.66. This is slightly under the common threshold of 0.70 (Hair et al., 2010) yet superior to the threshold of 0.6 (Nunnally, 1967). We also refer to the external validation below which shows that the construct behaves in line with previous research, and to the fact that in our analyses we could replicate prior findings of the relationship between TL and EE when not accounting for HPWS, all in further support of a satisfactory reliability.

### 4.3. Independent variables

High-performance work systems was conceptualized as an individual-level variable and measured based on Sun et al. (2007). We replaced their measures of performance appraisal with two items from Lepak and Snell (2002) as performance evaluation in the present study was likely to depend on judgment rather than objective criteria. We also added a measure of the level of pay based on much other research on HPWS (e.g., Lepak and Snell, 2002; Zhang et al., 2013). Finally, to avoid direct reference to leadership behaviors we removed the measures of “employee participation” (Sun et al., 2007) from the present analysis. Thus, based on a combined consideration of the best-loading items in their validation as well as good coverage of the construct domain, our measure included 17 items covering seven HPWPs: selection, training, pay, performance appraisal, career opportunities, job security, and job descriptions. Cronbach’s alpha of the scale was 0.85.

Transformational leadership was conceptualized as an individual-level variable focused on the middle-level supervisory manager’s behavior and measured by an abbreviated version of the scale developed by Podsakoff et al. (1990). We included nine items based on a careful consideration of the original validations and the construct domain in Podsakoff et al. (1990), Podsakoff et al. (1996), MacKenzie et al. (2001): three items for “core TL behaviors” and two items each for “high performance expectations,” “individualized consideration,” and “intellectual stimulation.” Cronbach’s alpha of the full scale was 0.88.

### 4.4. Mediating variables

Job control was measured with six items from van Yperen and Hagedoorn (2003) based on the factor loadings in Jackson et al. (1993). Cronbach’s alpha of the scale was 0.87.

Task related self-efficacy was measured using the four best loading items in Riggs et al. (1994), excluding the negatively worded or reverse coded ones. Cronbach’s alpha of the scale was 0.81.

OBSE was measured by three-items from Pierce et al. (1989). Cronbach’s alpha of the scale was 0.77.

Job demands was measured by three items capturing workload based on Bakker et al. (2004). Cronbach’s alpha of the scale was 0.76.

### 4.5. Control variables

Based on extant research we included controls for *gender*, *age*, and *tenure under the same superior*. Gender was measured as a dichotomous variable (0 = male, 1 = female). Age and tenure were measured as the number of full years.

### 4.6. The internal validity of our abbreviated scales

Table 1 exhibits the descriptive statistics of all variables, including their reliabilities and correlations. Due to our reduced measurement scales we examined both their internal and external validity in more detail. The reliability of our reflective measures was first assessed by calculating their Cronbach alphas, composite reliability, average variance extracted, and discriminant validity. As shown in Table 1, the scores for all constructs are above expected thresholds. All constructs also exhibit good judgmental qualities in terms of construct domain coverage, i.e., covering the important aspects in the original measurement instruments. This suggests good internal validity and domain coverage of our abbreviated scales (Stanton et al., 2002). For further internal validation, a confirmatory factor analysis (CFA) is presented below.

### 4.7. The external validity of our abbreviated measures

As for the critical aspect of the external validity of abbreviated scales (Stanton et al., 2002), we compared our correlations with those in previous research. In the case of our measures of TL and emotional exhaustion we find strong evidence of their external validity. First, in Kanste et al. (2007), in a study performed in the same cultural setting as the present one, the correlation between original comprehensive scales of both TL and emotional exhaustion was *r* = −0.15, exactly the same as in our study *r* = −0.15. Using similarly comprehensive scales, Gregersen et al. (2014) observed a correlation of *r* = −0.21. Hetland et al. (2007), Scheel et al. (2019) observed correlations of *r* = −0.12 and *r* = −0.22 (T1), respectively. All this provides evidence of satisfactory external validity of our abbreviated measures of TL and exhaustion. Harms et al. (2017) showed meta-analytic evidence of a slightly higher correlation of *r* = -0.23 between TL and emotional exhaustion, but this evidence is somewhat difficult to evaluate as it included a range of unpublished studies and a master thesis.

In the case of HPWS and emotional exhaustion, research is still both scarce and exhibiting much variation in findings, with Kroon et al. (2009), Zhang et al. (2013) finding no significant direct relationship, while both Fan et al. (2014), Kloutsiniotis and Mihail (2020) finding correlations of between *r* = −0.33 and −0.36. The correlation in our study is *r* = −0.27, which corresponds to Salas-Vallina et al. (2020) who reported a correlation of *r* = −0.26 using a partly different HRM construct and a two-wave study. Finally, the correlation between our measure of job demands and emotional exhaustion is *r* = 0.61. In Alarcon (2011) the weighted mean correlation was *r* = 0.49 with a 95% confidence interval of 0.45–0.52, whereas in Lee and Ashforth (1996) this correlation was *r* = 0.65 with a 95% confidence interval of 0.55–0.75. Thus, the correlation in our study is high, but not unprecedentedly high and well in line with the meta-analysis by Lee and Ashforth (1996).

Thus, overall extant research offers support also for the external validity of our measures (Stanton et al., 2002).

### 4.8. Analytical procedures

Our data was nested, with 308 employees nested within 76 teams. Therefore, we first calculated the ICC values for our main variables (Raudenbush and Bryk, 2002; see Table 1). Results indicate rather small ICC values for our outcome variables (<0.1), while 18% of the variance in TL and in HPWS could be attributed to team membership. Since low ICC values can bias effects on the between level (Preacher et al., 2010) and since we did not hypothesize team level effects, we decided to focus on an individual-level analysis in which we account for the nested structure of our data (Muthén and Satorra, 1995). Using Mplus version 8 (Muthén and Muthén, 2018), we carried out a confirmatory factor analysis (CFA) with maximum likelihood robust estimation. In this CFA we used all items for the independent, mediating and dependent variables. TL was conceptualized as a second-order factor in which the four leadership domains represent the first-order components. HPWS was also conceptualized as a second order construct. However, to account for the formative character of HWPS (Jiang et al., 2012; Hauff, 2021), we specified only the seven first-order components which we afterward summarized to an additive index (i.e., we conceptualized HPWS as a reflective-formative second-order construct). The model exhibited adequate fit based on Williams et al. (2009): χ^2^(863) = 1404.893, *p* < .001; CFI = 0.903; RMSEA = 0.045; SRMR = 0.064. In light of these results, to simplify the model and to avoid problems with model convergence, we computed the arithmetic means of all reflective constructs and used them in the path analysis (e.g., Den Hartog et al., 2013; Ward et al., 2022).

To examine the potential common method variance (CMV) in our data we tried a CFA with a model including an unmeasured latent method construct (ULMC) (Podsakoff et al., 2003). This model was not identified due to the complexity of the model. Thus, we could not evaluate the presence of CMV. However, although debated, recent evidence suggest CMV is unlikely to be the commonly understood threat for validity (Bozionelos and Simmering, 2022) and, most importantly, CMV cannot explain the *differential* effects of TL and HPWS which the hypotheses in this paper focus on. We return to the adequacy of our data for testing our hypotheses in the limitations section.

## 5. Results

**FIGURE 2:**
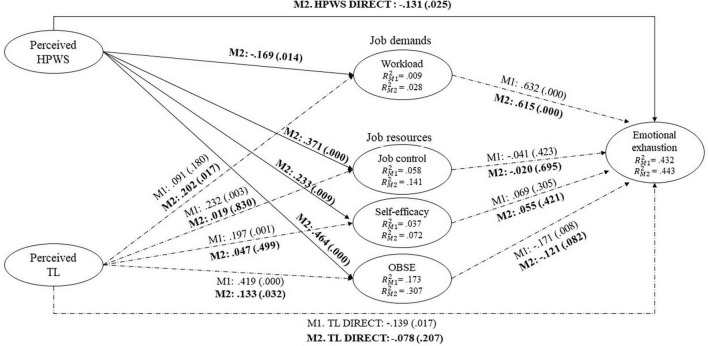
Model of direct relationship. M1: model 1–baseline model, Tl only. M2: model 2 including also high performance work system (HPWS). Individual–level analysis accounting for the nested structure of the data. Including controls.

We first estimated our baseline model, Model 1 [M1, χ^2^(3) = 6.295, *p* < 0.098; CFI = 0.992; RMSEA = 0.060; SRMR = 0.022], accounting only for TL, not HPWS (Figure 2). This confirmed the assumed positive relationships between TL and each of the resources (Figure 2) as well as both the direct (Figure 2) and total (Table 2) negative relationship between TL and emotional exhaustion, thus replicating much extant research on TL. However, M1 (Table 2) also showed that only one of the resources, OBSE, played a significant *unique* mediating role when simultaneously accounting for all three. The relationship between TL and job demands was insignificant in M1.

**TABLE 2:** Summary of the mediated and total relationships.

Model	Mediated and total relationships	Estimates and *p*-values
M1	TL → job demands → emotional exhaustion	0.058 (0.188)
M2	TL → job demands → emotional exhaustion	**0.120 (0.026)**
M2	HPWS → job demands → emotional exhaustion	−**0.104 (0.012)**
M1	TL → job control → emotional exhaustion	−0.009 (0.420)
M2	TL → job control → emotional exhaustion	0.000 (0.997)
M2	HPWS → job control → emotional exhaustion	−0.007 (0.696)
M1	TL → self-efficacy → emotional exhaustion	0.014 (0.328)
M2	TL → self-efficacy → emotional exhaustion	0.003 (0.613)
M2	HPWS → self-efficacy → emotional exhaustion	0.013 (0.425)
M1	TL → OBSE → emotional exhaustion	−**0.072 (0.015)**
M2	TL → OBSE → emotional exhaustion	−0.016 (0.224)
M2	HPWS → OBSE → emotional exhaustion	−*0.056 (0.069)*
M1	TOTAL direct and mediated TL → emotional exhaustion	−**0.148 (0.037)**
M2	TOTAL direct and mediated TL → emotional exhaustion	.029 (0.716)
M2	TOTAL direct and mediated HPWS → emotional exhaustion	−**0.286 (000)**

Significant relationships bolded. Weakly significant relationship in italics.

In our Model 2 [M2, χ^2^(3) = 10.128, *p* < .018; CFI = 0.985; RMSEA = 0.088; SRMR = 0.027], we included also HPWS. First, in line with H1a-c, M2 (Figure 2) shows that two of the significant relationships between TL and the mediators in M1 became insignificant once HPWS was taken into account: TL and job control (M1: β = 0.232, *p* = 0.003; M2: β = 0.003, p = 0.997), TL and self-efficacy (M1: β = 0.197, *p* = 0.001; M2: β = 0.053, *p* = 0.470), while the relationship between TL and OBSE was substantially reduced (M1: β = 0.419, *p* = 0.000; M2: β = 0.132, *p* = 0.032). Also the direct relationship between TL and emotional exhaustion became insignificant (M1: β = −0.139, *p* = 0.017; M2: β = −0.078, *p* = 0.207). However, the hypothesized mediated substitution effects of HPWS as proposed by H1a-c did not receive support (Table 2) much due to the insignificant *unique* resource-based effects on emotional exhaustion (Figure 2). However, the direct relationship between HPWS and emotional exhaustion was negative and significant (β = −0.131, *p* = 0.025). Further, M2 (Figure 2) also shows that the direct relationship between HPWS and job demands, when accounting for TL, was significant yet surprisingly *negative* (β = −0.169, *p* = 0.014) and Table 2 that job demands mediated a *negative* relationship between HPWS and emotional exhaustion (β = −0.104, *p* = 0.012). Equally surprisingly M2 (Figure 2) shows that the relationship between TL and job demands was *positive* and significant once we accounted for HPWS (TL M2: β = 0.196, *p* = 0.024) and Table 2 shows that this mediated a *positive* relationship between TL and exhaustion (TL M2: β = 0.120, *p* = 0.026). Therefore, while H2a and H2b were rejected based on the opposite hypothesized signs with respect to job demands, the mediating mechanism in the form of job demands remained valid. Finally, as shown in Table 2, in full support of H3 HPWS substituted the total direct and mediated negative effect on emotional exhaustion that would be attributable to TL if the latter was considered alone (HPWS total effect M2: β = −0.286, *p* = 0.000) whereas TL total effect M1: β = −0.148, *p* = 0.037 became in significant in M2 β = 0.029, *p* = 0.716. Finally, we note that most of the variance in emotional exhaustion was explained by job demands and that, despite the interesting significant effects, both TL and HPWS explained relatively little of the variance in job demands. We now turn to a discussion of these intriguing results.

## 6. Discussion

Against the backdrop of non-existent joint research on the relationships between TL, HPWS and health-related wellbeing, we set out to answer the research question: *Do the main effects of HPWS substitute the assumed main effects of TL on emotional exhaustion?* To answer this question, we used extant research to establish an assumed mediation model of both TL and HPWS, including a comprehensive set of mediators in the form of both resources and job demands in line with the JD-R model. We then put the explanatory power of TL and HPWS to a series of competitive tests derived on the basis of leadership substitutes theory. The results of our study are partly consistent with previous research, yet surprising and interesting on several accounts.

### 6.1. TL and wellbeing

Without considering HPWS, our study largely replicates extant research on TL that suggests it is positively related to all resources, job control/autonomy (Breevaart et al., 2014), self-efficacy (Gregersen et al., 2014) and OBSE (Kark et al., 2003) and also negatively related to emotional exhaustion (Arnold, 2017; Harms et al., 2017). This is important both for the credibility of our competitive contest between TL and HPWS theory (Gray and Cooper, 2010; Leavitt et al., 2010) and for establishing the substitution effect.

Importantly, when accounting for the role of HPWS, the relationships between TL and all the resources vanishes, as does the direct negative relationship between TL and emotional exhaustion. It is interesting that, when accounting for HPWS, the partial relationship between TL and job demands was highly positive, mediating an exhaustion-increasing effect of TL, even as the total relationship between TL on exhaustion was insignificant. All this suggests that the unaccounted effect of HPWS has led to an important omitted variable bias (e.g., Hill et al., 2021) in previous research on TL, both with respect to its resource provision (Kark et al., 2003; Breevaart et al., 2014; Gregersen et al., 2014; Arnold, 2017), and its effects on emotional exhaustion (Arnold, 2017; Harms et al., 2017). The substitution effects of HPWS that we identified also provide direct support for leadership substitutes theory (Jermier and Kerr, 1997), specifically the contention that we tend to over-emphasize the role of leader-centric interpersonal leadership behavior. Relatedly, our study provides some support for the general conjecture that to the extent leaders can influence, they can do so largely *“through technological, structural, and other impersonal processes in the organization”* such as HPWS, rather than purely by their *“superior-subordinate interactions”* such as TL (Jermier and Kerr, 1997: 98). It thus points to the importance of leaders engaging with the development and implementation of HRM practices (Steffensen et al., 2019), and supports the search for different sets of leader behaviors more directly akin to improve the deployment of HRM (Nishii and Paluch, 2018).

Beyond this, the marginal job demands increasing effect of TL that we found makes sense from the perspective that TL centers around providing employees with responsibility and pushing them to perform while also being theorized to provide resources (Arnold, 2017; Inceoglu et al., 2018). Based on our results it is the resource-improving power of TL that may be overestimated, whereas the pushing effect, and subsequent “high energy investment” leading to increased job demands may be underestimated (Inceoglu et al., 2018: 187; compare Fernet et al., 2015). Some further support for this is provided by Lin et al. (2020), however, only in the sense of increasing challenge stress with positive consequences for employees. To put this in further perspective it is also important to note that in our study the total effect of TL on emotional exhaustion was insignificant, presumably because its weak direct negative effect counteracted its tendency to increase job demands. It is also important to note that while the job demands increasing effect provides interesting support for the reasoning in Inceoglu et al. (2018) and interesting counter-evidence to research pointing to TL’s stress reducing effects (Harms et al., 2017), the explained variance in job demands was low.

### 6.2. Theoretical development in the case of TL

The results offer some more evidence for the importance of critically examining “*leadership tomfoolery*” (Bligh et al., 2011: 1,074) and specifically support arguments for the need to take TL theorizing back to the drawing broad (van Knippenberg and Sitkin, 2013). What we add to the latter study is concrete evidence of a lack of the expected causal influence of TL on a number of resources and emotional exhaustion, as no (positive) correlation implies no (positive) causality. More specifically, this supports the identified need to develop the theoretical and empirical understanding of the relationship between leadership, wellbeing and underlying resource-based mediating processes (Inceoglu et al., 2018). In line with their call for research on TL’s potential “wellbeing trade-off effects (p. 187) and our study, Vincent-Höper and Stein (2019) also question the positive resource-increasing and demands decreasing effects specifically of TL, as the latter found that there was little left of TL’s positive effects when controlling for health- and development-promoting leadership behavior. In this way our study, and indeed Vincent-Höper and Stein (2019), exemplify the fruitfulness of *“direct empirical comparisons [that] can help establish which theory is superior in a given domain and force more precision in the theoretical arguments for the theory that fares less well”* (Gray and Cooper, 2010: 629).

### 6.3. HPWS and wellbeing

First, the present study provides evidence of a negative relationship between HPWS and emotional exhaustion, corroborating recent research (Fan et al., 2014; Kloutsiniotis and Mihail, 2020; Wang et al., 2022; cf. Salas-Vallina et al., 2020). This offers further counter-evidence to conclusions that HPWSs *“do appear to negatively affect psychological and emotional health wellbeing”* (Jensen and Van De Voorde, 2016: 69; see also Han et al., 2020).

Second, the unique relationship between HPWS and *job demands* was negative in the present study, replicating the equally surprising results in Kloutsiniotis and Mihail (2020). Importantly, we note that in our study the negative relationship between HPWS and job demands became visible only once we accounted for TL, which suggests that not accounting for leadership may have led to important omitted variable biases (e.g., Hill et al., 2021) in research on HPWS and work intensification, corresponding to the inverse bias that our study points to in research on TL. This exemplifies that *“in our enthusiasm to find significant and theory-supporting results, scholars frequently fail to consider alternative explanations for a relationship”* (Leavitt et al., 2010: 660). In line with Kloutsiniotis and Mihail (2020), in our study the negative relationship between emotional exhaustion and HPWS is explained partly by its effect on reduced experienced job demands, but also by its direct reducing effect on emotional exhaustion. The mechanism of the latter remained unidentified in the present study. Together this emerging evidence calls into question the robustness and generalizability of theorizing (Han et al., 2020) and empirical research that has found HPWS directly and positively related to work intensification (Ramsay et al., 2000; Kroon et al., 2009; Jensen et al., 2013; Ogbonnaya and Messersmith, 2019; Qi et al., 2021; Zhu et al., 2022; see also Ehrnrooth and Bjorkman, 2012). Specifically, the change in the relationship between HPWS and job demands when accounting for TL in the present study was significant enough to shed some more doubt on the received view of the nature of this relationship (more on this in the next section). However, as in the case of TL, we note that even as this provides important counter evidence to much research on HPWS, the explained variance in job demands was low. This is important information also given that the explained variance is often not reported in research on HPWS and job demands (e.g., Kroon et al., 2009; Jensen et al., 2013; Kloutsiniotis and Mihail, 2020).

Third, our findings support the importance of simultaneously considering the influence of both HRM and leadership to arrive at more accurate estimates (Leroy et al., 2018). The study specifically contributes to the scarce simultaneous research on HRM, leadership and wellbeing by taking a different theoretical perspective on the independent effects of leadership and HRM. In our study TL did not interact with HPWS (post-hoc results not shown here). This is interesting in that other leadership styles (Kalshoven and Boon, 2012; Salas-Vallina et al., 2020; Wang et al., 2021; Hauff et al., 2022a,b) have been found to do that. Their interaction effects remains an important area of research, but so are their unique main effects as emphasized by our study, not least as the explanatory power of interaction effects tend to be marginal compared to the main effects, in particular when they are positive (e.g., Ehrnrooth et al., 2021; Hauff et al., 2022a,b).

### 6.4. Theoretical development in the case of HPWS

Overall, our results provide strong support for the call for more theorizing and research on the wellbeing effects of HPWS (Peccei and Van De Voorde, 2019; Han et al., 2020). Currently, we lack a theoretical explanation for the direct negative relationship between HPWS and experienced job demands, as also noted by Kloutsiniotis and Mihail (2020). The dominating assumption is that HPWS increases job demands (Han et al., 2020). More specifically, we need an explanation for why the main effects of HPWS on job demands/stress is sometimes negative (e.g., Kloutsiniotis and Mihail, 2020; the present study) and sometimes positive (Ramsay et al., 2000; Kroon et al., 2009; Jensen et al., 2013; Ogbonnaya and Messersmith, 2019; Qi et al., 2021; Zhu et al., 2022). One explanation may be omitted leadership variables, either in terms of their main effects (as in the present study) or as moderating substitutes (e.g., Wang et al., 2021). Scholars have also identified a range of other moderators explaining under what conditions HPWS is likely to intensify work and reduce wellbeing (e.g., Han et al., 2020). Future research should take a careful look at such moderators to see if general patterns can be found. Kloutsiniotis and Mihail (2020) also consider a curvilinear effect of HPWS as one explanation, but they note that the evidence for that is so far weak and conflicting. Below we suggest two other sets of possible explanations, one related to the ambiguity of the HPWS construct and one related to the ambiguity of its effects.

#### 6.4.1. The ambiguity of the HPWS construct

One possible explanation of the divergent findings is provided by the fact that the HPWS construct leaves room for different attributions as to its implementation (Van De Voorde and Beijer, 2015; Han et al., 2020). Relatedly, we suggest that HPWS may sometimes promote, or be moderated by, either collaborative mastery climates or competitive performance climates (Nerstad et al., 2018), wellbeing oriented or efficiency-oriented climates (Veld and Alfes, 2017), a predominant social exchange or economic exchange (Mihail and Kloutsiniotis, 2016). However, there is no generally accepted theory of how the HPWS construct *per se* promotes such outcomes. Different results with respect to work intensification and wellbeing in extant research may thus be due to such unaccounted aspects of HPWS. Omitted variables in research on HPWS may also include *other organizing principles*, beyond leadership (Collins and Smith, 2006). Such principles may in practice sometimes be implemented in combination with HPWS, and sometimes not, representing important independent main causes of increased job demands. One example may be up-or-out policies, most prevalent in consultancy firms and some academic institutions (“publish or perish”). Such policies could show up in experienced job security, but not all HPWS constructs include this as part of the construct.

#### 6.4.2. Resources and the ambiguity of HPWS effects

High-performance work systems may have inconsistent or paradoxical effects (Ho and Kuvaas, 2020), and specifically so on job demands and thus simultaneously both positive and negative effects on wellbeing (Han et al., 2020; Qi et al., 2021). However, the latter also points to the underlying complexity of the resource-based effects of HPWS, some resources possibly increasing job demands while some may reduce them. Indeed, even if our study questions the unique power of the focal resources to explain the effects of either TL or HPWS on exhaustion, the correlations weakly point to a possible paradoxical job demands-increasing effect of OBSE even as it reduces emotional exhaustion. We note that the relationship between specific resources and job demands has not been consistently evidenced in primary research on the JD-R model (Bakker and Demerouti, 2017). For example, Bakker et al. (2004) found a positive relationship between resources and job demands, while Xanthopoulou et al. (2007) found a negative one, using similar kinds of resource constructs. All of this highlights the need to further theorize the role of the resources that HPWS creates.

### 6.5. Practical implications

While it is often challenging for organizations to make sure that job demands are not too high (Goh et al., 2016), we provide evidence suggesting that the unique effect of HPWS is related to lower levels of experienced job demands and partly thereby to lower levels of emotional exhaustion, while the unique effect of TL is related to increased job demands. Our study thus clearly suggests that to support employee wellbeing organizations should invest scarce resources in HPWS development and implementation rather than TL. This provides actionable advice to top leaders, line managers and HR professionals, in line with recent research pointing to the importance of their involvement in HRM development and implementation (Nishii and Paluch, 2018; Steffensen et al., 2019). The importance of this prioritization represents novel information for organizational practice as positive leadership behavior in general, and TL behaviors specifically, have previously been understood as rather unambiguously enhancing employee wellbeing (Arnold, 2017; Harms et al., 2017) and as there are strong tendencies to attribute positive causality to leadership (Bligh et al., 2011).

### 6.6. Limitations

Carefully considering the limitations of a study is important (Aguinis and Edwards, 2014). First, while cross-sectional data even with lagged outcome data and/or multiple sources of data all essentially allow only for correlational conclusions, a specific limitation in the present study is our single-source data and related CMV-based endogeneity. However, scholars have recently argued that CMV is unlikely to be the commonly assumed threat to validity even as this remains debated (Bozionelos and Simmering, 2022). More importantly, our paper is focused on HPWS substituting the effects of TL, i.e., the relative importance of these relationships. Based on Podsakoff et al. (2003) CMV does not offer an explanation for the relative importance of independent variables. In fact, any *difference* between the effects of these variables is unlikely to be explained by CMV inflating the relationships since we have found no reason to believe that CMV would be systematically different in the HPWS-employee attitude and SL-employee attitude relationships, in particular that it would be stronger for HPWS. Also, by simultaneously considering HPWS and TL we address one important cause of endogeneity in both research streams, i.e., that of omitted variable bias (Antonakis et al., 2010; Hill et al., 2021). Noting that correlational studies can still “have important implications for future research” (Antonakis, 2017: 15), we argue that the present study is a case in point as it shows evidence for important substitution effects of HPWS, a related methodological contribution to theory contesting research, and points to surprising results that should inform the *“theory building process”* (Gray and Cooper, 2010: 620, 621) with respect to both TL and HPWS. Concerning causality, Scheel et al. (2019) also partially question the received relationship between TL and exhaustion, finding some evidence for an indirect reverse causal relationship. This is an excellent example pointing to the possibility that we need to revise much existing understanding in the field of management once scholars engage more broadly in longitudinal research, and in including important omitted variables. Thus, while the absolute correlations in this study may be biased upward and causality cannot be established due to our cross-sectional research design, we submit that our findings provide important information for future longitudinal research (Inceoglu et al., 2018; Peccei and Van De Voorde, 2019), suggesting that such research may remain substantially biased if not including both HRM and leadership in the model, even when primarily focused on one or the other.

Second, we did not analyse differential effects of various individual HPWPs (Guerci et al., 2022), various combinations thereof (Ogbonnaya and Messersmith, 2019; Villajos et al., 2019), nor did we consider dimensional effects of TL (Arnold, 2017). We also did not examine moderation effects of HPWS and various contextual elements (e.g., Jensen et al., 2013; Haider et al., 2020; Wang et al., 2022; Pahos and Galanaki, 2022; Zhang et al., 2022) as we were theoretically interested in a head-on comparison of the main effects of TL and HPWS. Finally, and importantly, we note that we did not hypothesize that HPWS would mediate the effects of TL. We find it crucial that such mediation is not what neither mainstream leadership theory, nor specifically TL theory, implies as these theories focus on the direct effects of the leader’s interpersonal behaviors. These behaviors are not focused on the development or implementation of structural aspects of the work environment such as HPWS, even if this may be an important task of leaders (Fischer et al., 2017). Vice versa, we also see nothing in the mainstream HRM constructs that would clearly causally influence specific supervisory leadership behaviors, for example TL. However, the internal relationships between HRM and leadership certainly deserve more theoretical attention (Leroy et al., 2018).

Finally, as our analyses relied on one database in a single country, the results of the present study need to be replicated/corroborated in different contexts, in addition to being extended by considering combinations of other leadership styles, HRM systems, mediators and outcomes.

## 7. Conclusion and future research

In summary, while we join others in pointing to the importance of longitudinal research designs in research on both HPWS (Peccei and Van De Voorde, 2019; Han et al., 2020) and TL (Arnold, 2017; Inceoglu et al., 2018), and expanded conceptualizations of HRM (Guest, 2017), our study suggests that all such efforts can arrive at more correct results by simultaneously considering both HRM and leadership. Further, the results of the analyses in the present study support the need for theoretical development of the wellbeing effects of both TL (Arnold, 2017; Harms et al., 2017; Inceoglu et al., 2018) and HPWS (Peccei and Van De Voorde, 2019; Han et al., 2020). More generally, we join calls for more theory contesting research (Gray and Cooper, 2010) that considers alternative explanations (Leavitt et al., 2010) to better understand when and for what wellbeing outcomes any leadership style may provide the better explanation, and when HRM systems may do so. A specific interesting suggestion is to compare the effects of the novel construct of health- and development-promoting interpersonal leadership behavior (Vincent-Höper and Stein, 2019) to HRM. It would also be important to study HRM and leadership as predictors of group or team wellbeing, not only because teamwork is increasingly prevalent and leadership may be relatively more important for team-level outcomes, but also because individual work-related wellbeing may in collectivist cultures be more dependent on the wellbeing of the group. Further, reverse causality is an interesting possibility. Good performance may contribute to the wellbeing of employees, increase their sense of resources and decrease the experience of job demands. Thus, the role of performance as a possible mediator of the effects of HRM and leadership on wellbeing is an interesting research question that also deserves attention in future research. Finally, while our data does not suggest non-linear relationships of either TL and HPWS (post-hoc tests not shown here), it does not exclude the plausibility of such a relationship in different contexts (Ho and Kuvaas, 2020). Also, this merits further research.

## Data availability statement

The raw data supporting the conclusions of this article will be made available by the authors, without undue reservation.

## Ethics statement

Ethical review and approval was not required for the study on human participants in accordance with the local legislation and institutional requirements. Written informed consent for participation was not required for this study in accordance with the national legislation and the institutional requirements.

## Author contributions

SH functioned as an additional content and methods expert, reviewing, writing, commenting, and carrying out the final analyses for the manuscript. All authors contributed to the article and approved the submitted version.
